# Prolonged intralymphatic delivery of dendritic cells through implantable lymphatic ports in patients with advanced cancer

**DOI:** 10.1186/s40425-016-0128-y

**Published:** 2016-04-19

**Authors:** Michal Radomski, Herbert J. Zeh, Howard D. Edington, James F. Pingpank, Lisa H. Butterfield, Theresa L. Whiteside, Eva Wieckowski, David L. Bartlett, Pawel Kalinski

**Affiliations:** Department of Surgery, Division of Surgical Oncology, Hillman Cancer Center, UPCI Cancer Pavilion, Suite 400, 5150 Centre Avenue, Pittsburgh, PA 15213-1863 USA; University of Pittsburgh Cancer Institute, Hillman Cancer Center, UPCI Cancer Pavilion, Suite 500, 5150 Centre Avenue, Pittsburgh, PA 15213-1863 USA; Department of Medicine, Hillman Cancer Center, UPCI Research Pavilion, Suite 137, 5117 Centre Avenue, Pittsburgh, PA 15213-1863 USA; Department of Immunology, Hillman Cancer Center, UPCI Research Pavilion, Suite 1.46, 5117 Centre Avenue, Pittsburgh, PA 15213-1863 USA; Department of Pathology, Hillman Cancer Center, UPCI Research Pavilion, Suite 132, 5117 Centre Avenue, Pittsburgh, PA 15213-1863 USA; Department of Otolaryngology, Hillman Cancer Center, UPCI Research Pavilion, Suite 132, 5117 Centre Avenue, Pittsburgh, PA 15213-1863 USA; Department of Infectious Diseases and Microbiology, Hillman Cancer Center, UPCI Research Pavilion, Suite 1.46, 5117 Centre Avenue, Pittsburgh, PA 15213-1863 USA; Department of Bioengineering University of Pittsburgh, Hillman Cancer Center, UPCI Research Pavilion, Suite 1.46, 5117 Centre Avenue, Pittsburgh, PA 15213-1863 USA

**Keywords:** Dendritic cells, Adoptive cell therapies, Intralymphatic port, Lymphatic vessels, Cannulation, Colorectal cancer, Immunotherapy, Human T cells

## Abstract

**Background:**

The currently-used modes of administration of immunotherapeutic agents result in their limited delivery to the lymph nodes and/or require repetitive ultrasound-guided nodal injections or microsurgical lymphatic injections, limiting their feasibility. Here, we report on the feasibility and safety of a new method of long-term repetitive intralymphatic (IL) infusion of immune cells, using implantable delivery ports.

**Methods:**

Nine patients with stage IV recurrent colorectal cancer underwent complete resection and received autologous dendritic cells (DCs) loaded with killed autologous tumor cells, KLH and PADRE, for up to four monthly cycles. Leg lymphatic vessels were cannulated, connected to 6.6Fr low-profile implantable subcutaneous delivery ports, and used to infuse 12 doses of DC over each 72 h-long cycle (every 6 h), followed by heparin flushes of the cannula-port system (one 72 h-long cycle per month). The patients who opted for alternative route of vaccine administration (2 patients) or whose ports became non-functional between cycles, continued treatment via intranodal (one injection/cycle) or intradermal (four injections/cycle) routes.

**Results:**

A total of nine lymphatic cannulations and implantations of subcutaneous delivery ports were attempted in seven patients, with a success rate of eight out of nine (89 %). The average patency of the IL delivery system was 7.5 (±3.2) weeks. All six patients with IL ports successfully completed at least one complete 72 h-long DC infusion cycle (12 injections). Five patients (56 %) completed two full IL cycles (24 IL injections). No patients received more than two IL cycles without replacement of the IL port, due to catheter occlusion and/or local side effects: cellulitis and hematoma. Intranodal and intradermal backup options were used in, respectively, one and two patients. Overall cohort survival was >28 (±25) months. One patient with aggressive recurrent carcinomatosis, who received DC vaccines by intranodal route is alive at > 90 months, without evidence of disease.

**Conclusions:**

We conclude that an intermediate-duration IL delivery of multiple doses of immunotherapeutic factors using implantable delivery ports is feasible, highly-tolerable and can be reproducibly performed in cancer patients to administer immune cells, or potentially, other immune factors. However, long-term IL port placement (>7.5 weeks), is not a currently-feasible option.

**Trial registration:**

NCT00558051, registered Nov. 13, 2007.

## Background

Several forms of immunotherapy have recently proven their clinical effectiveness in different forms of cancer, but the preferred mode of delivery of immunotherapeutic agents, including cellular products, allowing their optimal anti-cancer effects and minimal systemic toxicity remains unknown. In particular, systemic delivery of cellular therapeutic agents, such as Provenge, or adoptively transferred T cells results in preferential retention of such cells in liver, and lungs with very rapid loss of cells over time, which requires systemic cytokine support and/or myeloablation regimens. Similar, the delivery of therapeutic dendritic cell (DC) vaccines [1, 2] via subcutaneous or intradermal routes allows only very modest fraction of DCs (0.1–2 %) to enter the lymph nodes (LN). Moreover, the relatively long transit times of subcutaneously injected DCs [3, 4] can result in reduced ability of DCs to produce desirable type-1 cytokines and chemokines by long-term activated DC [5, 6].

In an attempt to overcome these problems, early protocols involved the injection of very high numbers of DCs (10^7^–10^8^ cells) presenting challenges with generating adequate numbers of cells, high costs and logistic challenges. Direct intranodal injection under ultrasound guidance has been utilized to deliver vaccine to the secondary lymphoid organs, but such method is technically challenging, often leads to the counterproductive DC accumulation in perinodal fat tissue and lymph node damage [7]. Direct intralymphatic (IL) injection of DCs using repeated temporary cannulations, similar to the method traditionally used in lymph-node mapping, has been successfully tested in a small number of patients [8, 9]. However, intermittent dosing with large “pulse” doses of DCs via the IL route does not mirror a physiological response nor is it feasible to perform repetitively, due to scar formation, high amount of personnel time and logistic challenges.

To increase the feasibility of repeated intralymphatic delivery of DCs (and potentially other factors such as T cells, stimulatory cytokines or checkpoint blockers) over multiple treatment courses, we tested the feasibility of using implantable ports, such as those used for intravenous access, to avoid the need for related lymphatic cannulations. We developed a modification of the method originally used to collect the outflowing lymph over periods of up to 10 days [10–13]. The key modifications involved a semi-permanent implantable subcutaneous delivery port to allow multiple injections and limit the infection risk, and heparinization of the port/catheter system to preserve its long-term patency in the absence of continuous lymph outflow. Herein, we report on the feasibility and limitations of implanting such lymphatic access ports in advanced cancer patients undergoing experimental DC therapy.

## Results

### Feasibility of intralymphatic port placement and duration of port patency

A total of nine patients with stage IV CRC underwent R0 resection (Table 1). The average age of the cohort was 51 (± 11.25) years. The cannulation was not attempted in two patients, who, for logistic reasons, started the experimental treatment using intranodal or intradermal delivery routes.

**Table 1:** Patient demographics and disease

Patient	Primary tumor	Resected metastatic site for generation of vaccine	Time from initial diagnosis of disease to vaccine treatment (wks)	Time from diagnosis of stage IV disease to vaccine treatment (wks)	Number of previous surgical treatments for disease	Previous chemotherapy regimens
1	Colon	Liver	30	21	2	FOLFOX FOLFIRI Bevacizumab Capecitabine
2	Colon	Peritoneum, Liver	161	161	4	XELOX XELIRI Bevacizumab
3	Colon	Peritoneum	119	119	2	FOLFOX FOLFIRI Bevacizumab
4	Rectal	Peritoneum, Liver, Lymph Nodes	374	282	6	FOLFOX FOLFIRI Cetuximab Capecitabine
5	Colon	Peritoneum, Colon	161	161	3	FOLFOX FOLFIRI Bevacizumab
6	Colon	Peritoneum	152	152	3	FOLFOX FOLFIRI Bevacizumab Capecitabine
7	Colon	Pancreas, Peritoneum	208	208	5	FOLFOX FOLFIRI Cetuximab Bevacizumab Capecitabine
8	Rectal	Peritoneum, Small Bowel, Lymph Nodes	344	150	4	FOLFOX FOLFIRI Capecitabine
9	Rectal	Peritoneum, Liver	163	141	3	FOLFOX FOLFIRI Bevacizumab

**Fig. 1:**
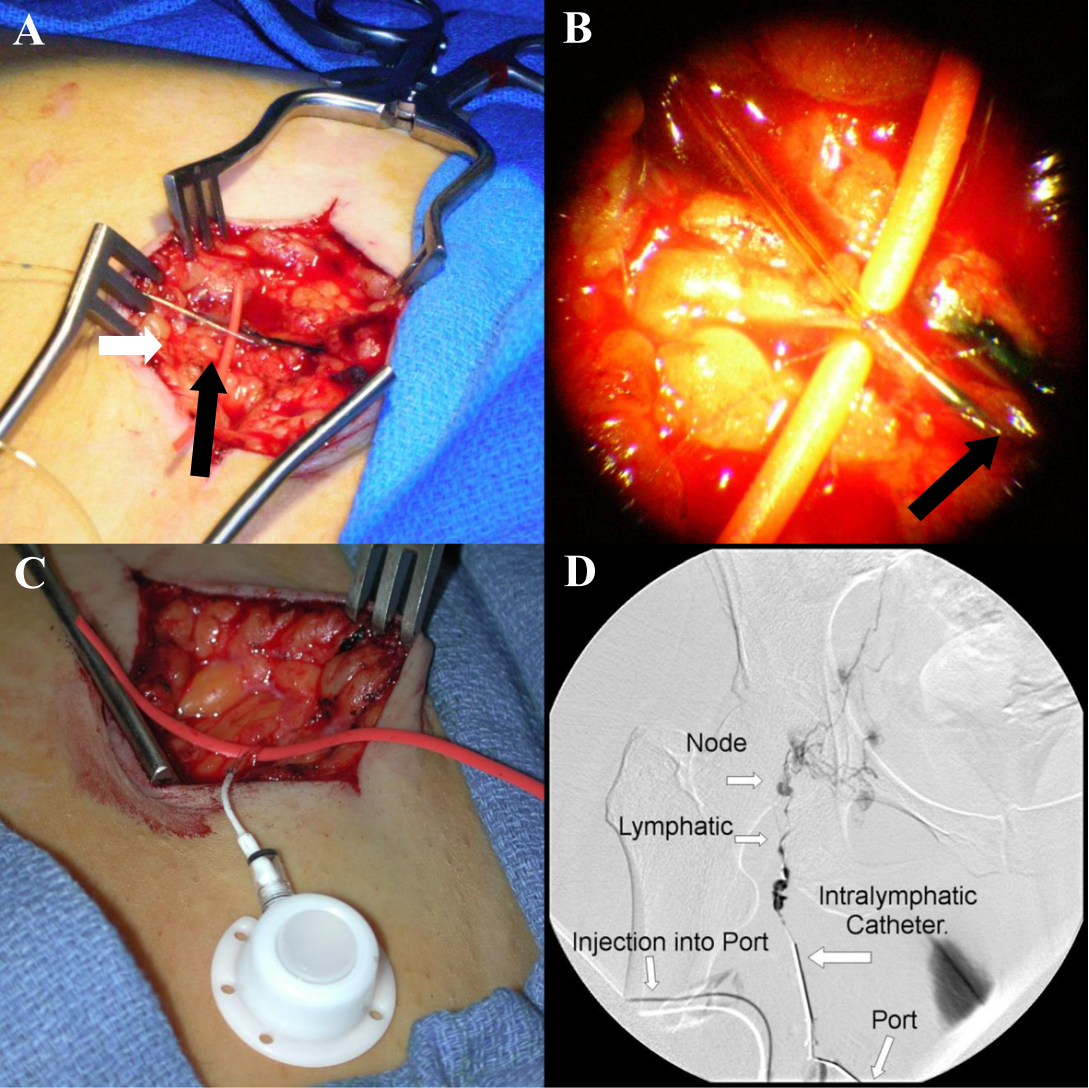
Operative steps for intralymphatic cannulation. **a**) Cut down over the femoral vessels. A vessel loop is used to encircle the femoral lymphatic vessel (white arrow) and after sharp sharp incision of the lymphatic vessel, the cannula is threaded (black arrow) using an operative microscope **b**) View through operative microscope of the cannula entering intralymphatic vessel (dark arrow) **c**) Intralymphatic port (connected to a lymphatic vessel) prior to its implantation in the subcutaneous pocket **d**) Lymphangiogram demonstrating patency of a subcutaneous intralymphatic port. Contrast material (2 cc) is seen flowing into the right femoral lymphatic vessel via a subcutaneous port and accumulating in multiple inguinal lymph nodes

**Fig. 2:**
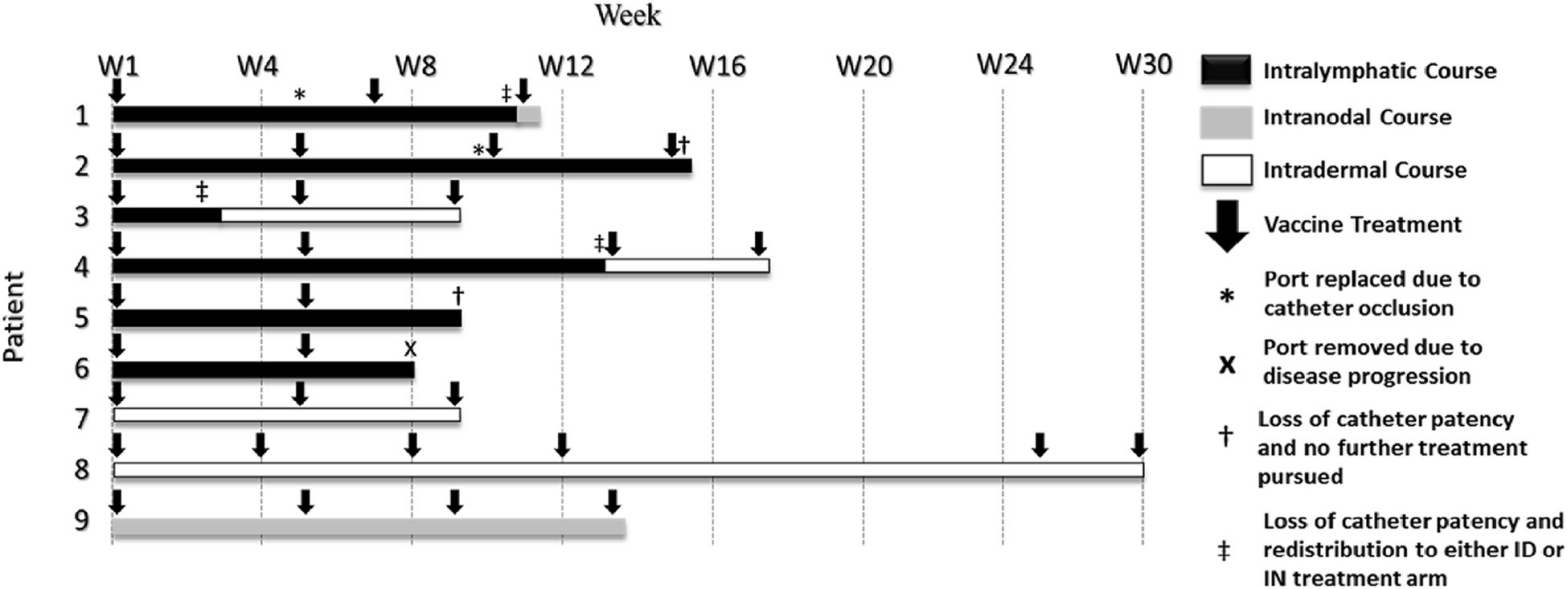
Treatment schema and duration of the intralymphatic catheter patency in the individual patients. *Black*: Duration of treatment involving intralymphatic cell delivery; *Grey:* Intranodal delivery; *White:* Intradermal delivery. Arrows represent the timing of the individual courses of treatment (12 doses over 72 h of each course of intralymphatic cell delivery; 3 doses over 72 h per course of intradermal cell delivery; single injections per each course of intranodal delivery)

Six of the remaining seven patients successfully received IL ports. Only one patient’s lymphatic system could not be cannulated. Two patients were successfully re-cannulated following the occlusion of the initially functional ports. A total of nine IL ports were attempted to be placed in a total of seven patients (including replacement ports in 2 patients) and a total of eight of nine attempted cannulations and port implantations were successful, corresponding to 89 % success rate (Figs. 1a-1c, and Fig. 2).

Port patency was confirmed at the time of implantation via lymphangiogram (Fig. 1d). Ports were flushed weekly with heparinized saline to prevent occlusion and confirm patency. Average port patency was 7.5 (± 3.2) weeks (Table 2). All patients who received a port had at least 1 course of IL vaccine. Five patients (56 %) successfully received at least 2 concurrent courses of IL vaccine (patients 1, 2, 4, 5, 6). No patients received more than 2 concurrent courses of IL vaccine without having the port replaced due to catheter occlusion or study withdrawal secondary to disease progression (patient 6). Three of the six patients (50 %) who received IL catheters received only IL administered vaccine (patients 1, 5, and 6). The most common complications encountered were loss of catheter patency, which occurred in 6 of 7 instances (patients 1-twice, 2, 3, 4, and 5, 86 %) followed by cellulitis occurring in 3 (43 %) of 7 instances (Table 2). One patient, #2, had recurrent cellulitis diagnosed at 1, 5, and 10 weeks and treated on an outpatient basis with oral antibiotics. Two patients (5 & 6, 33.3 %) had symptoms of flushing at the time of vaccine infusion which resolved after cessation of the infusion. One patient (16.6 %) had a small infected hematoma that was treated with oral antibiotics (patient 1). Other self-limited side effects reported were erythema at the injection site, fatigue, and diarrhea.

**Table 2:** Total vaccine delivered, side effects, and complications

Patient	No. of Treatment Courses	No. of DCs delivered (cells)	Side effects	Complications	
1	3	6x10^6^	None	Complication	Time of Complication (wks)
				Loss of Catheter Patency	5
				Loss of Catheter Patency	11
				Hematoma with Cellulitis	10
2	4	8x10^6^	None	Complication	Time of Complication (wks)
				Cellulitis	1, 5, 10
				Loss of Catheter Patency	10
				Loss of Catheter Patency	16
3	3	6x10^6^	None	Complication	Time of Complication (wks)
				Cellulitis	2
				Loss of Catheter Patency	3
4	4	8x10^6^	Erythema	Complication	Time of Complication (wks)
				Loss of Catheter Patency	13
5	2	4x10^6^	Flushing during administration	Complication	Time of Complication (wks)
				Hematoma	5
				Loss of Catheter Patency	9
6	2	4x10^6^	Flushing during administration	None	
7	3	4.9x10^6^	None	None	
8	6	11.8x10^6^	Erythema	Complication	Time of Complication (wks)
				Failed Catheter Placement	0
9	4	8x10^6^	Fatigue, Diarrhea	None	

### DTH

No patients had an initial positive DTH reaction to tumor lysate, KLH, or PADRE peptide. Patient 5 had a minimal positive DTH reaction to KLH at 6 weeks measuring 3 x 4 mm while patient 6 had the strongest positive DTH reaction to KLH at weeks 6 and 14 measuring 33 x 40 mm and 48 x 45 mm, respectively (Table 3).

**Table 3:** Survival and DTH Responses

Patient	Survival from initiation of vaccine	DTH response	IL-12p70 Production (pg/mL)
1	27 months	No Reaction	931
2	9 months	No Reaction	N/A
3	7 months	Positive at week 6 to KLH	2067
4	22 months	Positive at weeks 6 and 14 to KLH	N/A
5	15 months	No Reaction	341
6	13 months	No Reaction	1134
7	> 90 months^a^	No Reaction	N/A
8	28 months	No Reaction	1714
9	50 months	No Reaction	3937

^a^Patient alive without evidence of disease; N/A: Data not available

### Survival

**Fig. 3:**
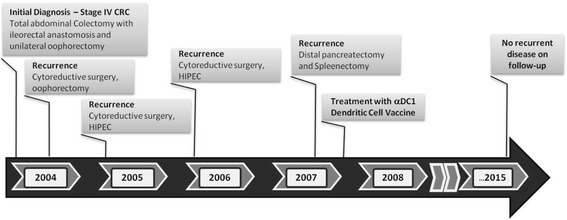
Clinical course of the disease and previous treatments of Patient # 7, the remaining long-term survivor without evidence of recurrent disease. That patient with high-level of microsatellite instability had three prior resections of the repetitively recurring intraperitoneal tumor, but remains without any sign of disease recurrence >90 months following the fourth resection, which was combined with intranodal DC vaccine administration

The average time to progression from the administration of vaccine was 3.4 (± 2.1) months. The average survival for the cohort after the initiation of vaccine was 28 (± 25) months. Of the 9 patients, 1 patient (7.5 %) is still alive (greater than 90 months after the initial DC treatment date, 128 months after initial diagnosis of Stage IV disease). Of interest, that patient had a history of recurrent carcinomatosis and four prior resections due to recurrent disease, but is still alive without evidence of disease at >90 months after the fourth surgery and intranodal DC administration (Fig. 3). She received a total of 4.9x10^6^ cells via 3 intranodal injections.

No correlation was observed between amount of vaccine given and time to progression or survival in the overall treated cohort.

## Discussion

We evaluated the feasibility and safety of semi-continuous intralymphatic infusions as a means of continued delivery of human cells, and potentially other factors to the lymph nodes of cancer patients. Short-term intralymphatic cannulation has been routinely used in imaging studies (regional lymph node mapping) and has been previously used for dendritic cell delivery. However, repeated cannulations are not feasible in routine clinical practice due to logistic and cost considerations, as well as increased risk of infections, fibrosis, and damage to neurovascular structures and as well as patient discomfort. While prolonged cannulation of lymphatic vessels to collect human lymph and associated cells has been successfully used in several studies, the feasibility of repeated access of lymphatic vessels in the absence of continued lymph flow has not been tested.

Our current results demonstrate that even one month-long lymphatic cannulations (up to 7.5 weeks) are feasible and can be reliably performed to deliver multiple doses of human immune cells, using implantable subcutaneous delivery ports. While our current data does not support immunologic or clinical advantage of using this route to deliver dendritic cell vaccines (our longest-surviving patient received exclusively intranodal DC injections), it remains to be tested if the method described can be used to administer other types of immune cells, checkpoint blockers or immunostimulatory cytokines, to increase their effectiveness and limit systemic toxicities. One potential application is the adoptive transfer of tumor-specific T cells, which are typically retained in lung and liver and show very short half-life following intravenous administration [14] to promote immune memory and sustained therapeutic effects.

Despite our initial concerns that the patency of the intralymphatic ports in the absence of lymph flow will require maintaining the catheter flow using low-rate saline infusion (which was also effective, clinicaltrials.gov NCT00390339) due to the presence of fibrin and the documented ability of lymph for form clots [15]. We observed that it can also be maintained by weekly heparin flushes, or an overage period of 7.5 weeks. In contrast, in none of the patients we managed to maintain the patency of the system for longer than 8 weeks, due to recurrent complications and port occlusion. The most frequent complication was port occlusions. These complications may be prospectively alleviated by designing a T-tube type catheter which would cannulate the lymphatic vessel both proximally and distally and thus allow flow through the device.

We also observed a high rate of cellulitis in the areas of intralymphatic port implantation, which phenomenon might have been promoted by the presence of a foreign body in an area of inhibited lymphatic flow, thus causing a localized inflammatory reaction. Additionally, ports were placed in the groin which may have facilitated a high infectious risk, as it is known that femoral central venous catheters have a high infectious risk over central venous catheters placed in other areas of the body [16]. However, all cases of cellulitis were controlled with oral antibiotic therapy and we did not observe formation of abscesses, positive cultures, or blood born infections.

To date, no studies have examined the long-term use of intralymphatic ports as a delivery mechanism. A study by Juillard et al. showed the feasibility of repeated monthly intralymphatic injections of irradiated tumor cells at a concentration (1x10^7^ cells per mL) in 21 patients with various advanced malignancies [17]. Another study performed by Lesimple et al. evaluated 14 patients with metastatic melanoma who received ex vivo matured dendritic cells pulsed with tumor peptides on a monthly basis. Study participants received the initial dose of vaccine via a single intralymphatic injection in the foot, followed by intranodal injections at 4 and 8 weeks [18]. Grover et al. delivered high doses (2x10^8^) autologous dendritic cells loaded with melanoma associated peptide antigens in 6 patients, using repeated lymphatic cannulation [19].

While our study demonstrates the feasibility of prolonged cannulation of human lymphatic vessels, it does not provide an indication of an advantage of this route to deliver DC vaccines. The two long-term survivors in our study had either intranodal or intradermal delivery of vaccine and all but one patient in the study cohort had progression of their underlying disease. Even with aggressive disease (three prior resections of the repetitively recurring intraperitoneal tumor), patient 7 in our study had no recurrence after administration of the vaccine. Interestingly, this patient had a high level of microsatellite instability that may be uniquely responsive to immunotherapy [20]. Despite a good clinical outcome, contrary to our expectations, this patient had a negative DTH response to all antigens tested.

## Conclusions

Our data demonstrate that prolonged intralymphatic port placement (up to 7.5 weeks) is a feasible option to repetitive (re)cannulations of lymphatic vessels to repetitively administer DCs or other factors. In contrast, but long-term intralymphatic port placement is not a currently feasible option due to port complications (occlusion and cellulitis). Although the current study did not provide us with any indications of a potential advantage of using this route to administer DC vaccines, the feasibility of cannulating lymphatic vessels over multiple days and weeks may provide an alternative to the current routes of delivery of other types of immune cells or immunotherapeutic drugs.

## Methods

### Patient population and study design

A total of 9 patients were enrolled in the study under the University of Pittsburgh IRB-approved protocol UPCI 05–063 (NCT00558051), following informed consent. Inclusion criteria consisted of ECOG performance status of 0 through 2, age greater than 18, platelet count greater than 100000 per μL, hematocrit greater than 27, white blood cell count greater than 2000 per μL, creatinine and bilirubin levels less than or equal to two-times the upper limit of normal, adequate recovery from all side effects of their previous therapy, no chemotherapy, radiotherapy, major surgery, or biologic therapy for their malignancy in the 2 weeks prior to the vaccine administration, and patients must undergo surgical resection of metastatic colorectal cancer to minimal evidence of disease (R0 or R1 status). Exclusion criteria included recent (less than 2 weeks) immunosuppressive treatment, including steroids, uncontrolled pain, active autoimmune disease, positive serology for hepatitis B, C, or HIV, allergy to heparin or local anesthetic, concurrent additionally malignancy, and pregnancy.

Patients received DCs by one of three routes: intralymphatic, intradermal or intranodal. The intralymphatic route was prioritized, with the intradermal or intranodal adminstration serving as backup options for patients who could not be cannulated, either due to the failure of (attempted) catheter implantation or due to logistics (without attempted cannulation), such as a patient’s reluctance to undergo inpatient treatment over 4 nights per course (needed for intralymphatic infusions).

All patients underwent an R0 resection and tumor cells were prepared and cryopreserved by the University of Pittsburgh Cancer Institute Immunologic Monitoring and Cellular Products Laboratory (IMCPL) until generation of the DC product. Patients in each group were considered evaluable if they have received 2 cycles of vaccine by any route. Patients whose intralymphatic delivery system lost patency could either be re-cannulated or continue to receive the DCs by alternative routes. Following the accrual of the three patients who each successfully completed at least two intralymphatic courses, the remaining patients got the option of starting the treatment using the backup routes. Therapy could be discontinued at any time under the following conditions: disease progression, intercurrent illness that prevents administration of vaccine, unacceptable adverse event(s), severe reaction to delayed type hypersensitivity (DTH) testing, patient withdrawal, dosing delays greater than 4 weeks, and negative changes in the patient’s general condition. Patients with remaining vaccine could receive additional courses of vaccination by any route.

### Leukapheresis and preparation of autologous tumor-loaded DCs

After recovery from surgery, each patient underwent limited 90 min leukopheresis. Monocytes were isolated from the pheresis product by the IMCPL, using the Elutra Cell Separation System, washed, and plated at a concentration of 0.5-1x10^6^ cells/mL in tissue culture flasks in therapeutic grade antibiotic free CellGro® Serum-free Media (CellGenix GmbH, Freiburg, Germany) with 1000 U/mL GM-CSF (UPCI Pharmacy, Pittsburgh, PA) and 1000 U/mL IL-4 (CellGenix GmbH, Freiburg, Germany) followed by incubation at 37 °C and 5 % CO_2_ [21]. After by 5–7 day cultures and were matured for 42–48 h in alpha-type-1 polarizing cytokine cocktail [21] consisting of IL-1β (25 ng/mL, CellGenix GmbH, Freiburg, Germany), TNF-α (50 ng/mL, CellGenix GmbH, Freiburg, Germany), IFNα (3000 U/mL, Merck Co, Whitehouse Station, NJ), poly-I:C (20 mg/mL, EMD Millipore, Philadelphia, PA) and IFNγ (1000 U/mL, InterMune Inc, Brisbane, CA) [21].

Fresh tumor tissues were minced and incubated with collagenase and DNAse, washed thrice with normal saline, UVB- and γ-irradiated with 20,000 Rads to induce apoptosis. Apoptotic tumor cells and either KLH protein (50 mg/mL, Biosyn Corp, Carlsbad, CA) or PADRE peptide (20 mg/mL, University of Pittsburgh Peptide Synthesis Facility, Pittsburgh, PA) were added to the autologous DCs (KLH- or PADRE-supplemented DC product was used in every second cycle of vaccination) at the time of the induction of maturation. Mature antigen-loaded DCs were harvested after 42–48 h, washed three times with sterile pH 7.2 PBS, and tested for sterility and other release criteria (endotoxin less than 5.0 EU/kg of body weight, DC viability > 70 %, CD83 > 70 % on CCR7 > 50 % on live DCs, less than 10 % contaminating CD3^+^, CD19^+^ or CD14^+^ cells. The ability of DCs to produce IL-12p70 following stimulation with CD40L-transfected J558 cells has been evaluated, as described before [5, 22].

### DC vaccine administration

Patients receiving intralymphatic infusions had a low profile implantable access port placed by cut down and cannulation of the femoral lymphatic vessel of either the right or left leg. In brief, a minimal volume of Isosulfan Blue (Covidien Inc, Mansfield, MA) was injected into the intradermal space 15 cm distally from the inguinal crease and massaged to facilitate lymphatic drainage. An incision was made 5 cm proximally to the dye injection site and dissection was carried down to the femoral lymphatic vessel which was isolated and encircled with vessel ties to obtain proximal and distal control. Using an operative microscope, the femoral lymphatic vessel was incised with a scalpel and cannulated with a 6.6 Fr BardPort MRI Low Profile Implanted Port (Bard Access Systems, Salt Lake City, UT) under direct vision. The catheter was secured with absorbable suture. Blunt dissection was used to make a subcutaneous pocket for the port at an easily accessible area of the thigh. A lymphangiogram was obtained to confirm placement and patency of the port. On days of administration of the IL vaccine, patients were admitted to the Clinical & Translational Research Center (CTRC) at UPMC Montefiore Hospital, Pittsburgh, PA. Ports were accessed in a sterile fashion and vaccine was delivered via slow infusion (0.5 mL over 1 min) followed by albumin injection (0.2–0.5 mL over 1 min) and a heparin flush (0.5 mL over 1 min). Vaccine, albumin, and heparin flushes were carried out every 6 h for a total of 96 h within each treatment course. Each course of intralymphatic treatment consisted of 16 divided doses of autologous tumor-loaded DCs totaling 2x10^6^ cells/course. Treatment courses were administered every 4 weeks. Patients that had port complications or were unable to have a port placed could receive the remaining DC product by either intranodal or intradermal injections.

Intradermal vaccinations were administered as 4 daily injections of autologous tumor loaded DCs (1 mL) administered over 4 days per course (2x10^6^ cells/course) on an outpatient basis. A single course of vaccine was administered every 4 weeks. Injections were in the vicinity of the major nodal basin of the thigh.

Intranodal vaccines were administered as single ultrasound-guided intranodal injections (1 mL) of autologous tumor loaded vaccine per course (2x10^6^ cells/course), in either the inguinal or axillary lymph node basins. A single course of vaccine was administered every 4 weeks. Delivery sites were rotated to avoid excessive lymph node damage and scar formation. Injected lymph nodes were between 5 and 20 mm in size.

### Patient- and port evaluation and surveillance

During the treatment period, patients had a complete physical examination with performance status on each day of vaccination. Attempted blood collections for in vitro analysis occurred at weeks 0, 2, 4, 6, 10, 14, 22, and 24. One patient had a repeat blood draw at 2 years. Patients with intralymphatic ports were followed by weekly evaluations and port flushes for up to 12 weeks, in addition to their routine follow-up for their underlying disease. The absence of occlusion of the port/intralymphatic delivery system was confirmed at each point by port flushes. All patients were evaluated 2 weeks after the last course of vaccination and then monthly for 5 months total. Lifelong follow-up consisted of patients being contacted every 3 months within the first 3 years post-treatment, every 6 months until year 5, then annually afterwards. Patients without evidence of disease progression were eligible for re-vaccination on a monthly basis starting at least 4 weeks after the previous vaccination.

### Delayed Type Hypersensitivity Testing (DTH)

Baseline DTH testing against the autologous tumor cell lysate and to the heterologous helper antigens KLH protein and PADRE peptides occurred on the first day of vaccine administration followed by subsequent tests at 6 and 14 weeks. Testing consisted of 100 μL intradermal injections of the autologous tumor cell lysate used in the vaccine, whole vaccine (both the PADRE and KLH containing variants), PADRE peptide (100 μg), and KLH protein (100 μg) at different sites. Skin tests were read at 48 h. A positive DTH response against antigen was defined as an increase of 3 mm of induration post dendritic cell vaccine over pre-dendritic cell vaccine. Vehicle (PBS at pH 7.4) was used as the negative control in both pre and post- dendritic cell vaccine evaluations.

## Abbreviations

Ag: antigen
CTL: cytotoxic T cell
DC: dendritic cell
DTH: delayed-type hypersensitivity
IFN: interferon
ID: intradermal
IL: intralymphatic
IN: intranodal
KLH: keyhole limpet hemocyanine
OS: overall survival
T_reg_: regulatory T cell
